# Supplementary material for: Do Negative Self-Evaluative Emotions Enhance Healthier Food Choices? Exploring the Moderating Role of Self-Affirmation
Source: Behav Sci (Basel). 2024 Jun 26;14(7):538. doi: 10.3390/bs14070538 (PMC11274215; doi:10.3390/bs14070538)
Supplement: Supplementary file 1 [file behavsci-14-00538-s001.zip › behavsci-2924043-supplementary.pdf]

Supplementary Materials

Registration form

Dear participant,

Welcome to our study. The study, conducted by the University of Groningen (www.rug.nl) in the Netherlands, is aimed at understanding individuals' dietary patterns.

Please leave your email address later to complete registration. Once you have successfully registered, you will receive a confirmation letter from us via email. To facilitate randomization process and data collection, please note that everybody should use your own email address to complete registration and you are not allowed to register by using someone else's email address. We guarantee that all information of the experiment will be used for research only, and the information you provide to us will be strictly protected. Last but not least, participating in the experiment is completely voluntary, and we hope that every participant who participates in the study will make decisions or answer questions truthfully and carefully. During the experiment, each subject has to pass an attention check. Participants who fail to pass the attention check will be asked to redo the experiment from the beginning. If you have any questions, please send an email to: j.li.32@student.rug.nl. If you agree with this study, please leave your email address here________

Online experiments' questionnaire

Control group:

1. Which one aspect is medium important to you?

There are always some aspects of our lives that are important to us and some aspects that are less important. Based on your own experience, choose the one aspect of your life that you think is medium important to you.

o Theory: Science, technology and research progress are important to me
o Economics: Practicality, money and things related to finance are important to me
o Aesthetics: Art, music and other art-related things are very important to me
o Social life: People, society, relationships and social order are important to me
o Politics: Leadership, domination and authority are important to me
o Religion: God, faith and religious practices are important to me
o Environment: Nature, the earth, animals and other species are important to me
o Hedonic: It's important for me to enjoy life and satisfy my desires

Theory:

1. If you are watching TV, there are two documentaries for you to choose from, which you will watch:

A. space exploration B. overseas investment

2. Which of the following do you think has made the most contribution to the progress of human society:

A. Newton, the founder of theoretical physics B. Martin Luther King, the champion of human rights

3. Which of the following areas do you think is more important for human beings:

A. mathematics and physics B. theology

4. If you are a professor and you have the necessary knowledge, you would like to teach:

A. literature and art B. physics and chemistry

5. If you see two articles in a newspaper of the same length, which one do you choose to read?

A. The political summit will beheld tomorrow B. Major scientific discovery

Economy：

1. In your opinion, the main influence of the European Union is:

A. facilitated free international trade B. formed a political bloc with strong influence in international affairs

2. Which of the following functions do you think is the most important for a modern leader:

A. Ensuring that goals are achieved B. Encouraging mutual respect among subordinates

3. If you saw two articles in a newspaper of the same length, which one would you choose to read?

A. Leaders of religious groups work together to promote unity

B. the international market environment has greatly improved

4. If you go to an exhibition, you tend to go to:

A. The latest electronic goods, such as home appliances and cars

B. Scientific research equipment, such as medical equipment

5. Which role of education do you think is more important:

A. Provide individuals with the competitiveness to increase their income level

B. Help individuals better integrate into society

Aethetics：

1. Do you accept that great artists such as Mozart, Shakespeare, and Picasso were selfish or even harmful:

A. Yes B. No

2. Which of the following questions would you like to discuss with others?

A. Movies from the '90s

B. European relations after the fall of the Berlin Wall

3. You are sick in bed on Sunday, it is you hear a radio program, you wish it was:

A. Pop music B. Religious programs

4. If you were given the chance to form a group of your own, what would you choose?

A. Political groups

B. Bands or other art groups

5. Current technological developments mean that we have reached a stage of social civilization that is more advanced than earlier civilizations such as ancient Greece:

A. Yes B. No

Social aspects：

1. Which of the following do you think has made the most contribution to the progress of human society:

A. Newton, the founder of theoretical physics B. Martin Luther

2. Do you accept that great artists such as Mozart, Shakespeare, and Picasso were selfish or even harmful:

A. Yes B. No

3. Which of the following functions do you think is the most important for a modern leader:

A. Ensuring that goals are achieved B. Encouraging mutual respect among subordinates

4. Would you like to donate to:

A. Missionary organizations B. Disaster relief groups

5. Which role of education do you think is more important:

A. Provide individuals with the competitiveness to increase their income level

B. Help individuals better integrate into society

Political aspects：

1. In your opinion, the main influence of the European Union is:

A. facilitated free international trade B. formed a political bloc with strong influence in international affairs

2. Which of the following questions would you like to discuss with others?

A. Movies from the '90s

B. European relations after the fall of the Berlin Wall

3. If you were given the chance to form a group of your own, what would you choose?

A. Political groups

B. Bands or other art groups

4. If you see two articles in a newspaper of the same length, which one do you choose to read?

A. The political summit will beheld tomorrow B. Major scientific discovery

5. Which of the following do you think deserves better recognition:

A. a world-class politician B. a Nobel Prize winner for literature

Religion：

1. The Bible, as a book, has great literary value over its religious value:

A. Yes B. No

2. Which of the following areas do you think is more important for human beings:

A. mathematics and physics B. theology

3. Which organization would you rather donate to:

A. religious communication working group B. refugee relief group

4. If you saw two articles in a newspaper of the same length, which one would you choose to read?

A. Leaders of religious groups work together to promote unity

B. the international market environment has greatly improved

5. You are sick in bed on Sunday, it is you hear a radio program, you wish it was:

A. Pop music B. Religious programs

Environments：

1. Which organization would you rather donate to:

A. The Society for the Protection of Animals

B. The Society for Disease Prevention

2. Which role of education do you think is more important:

A. Provide individuals with the competitiveness to increase their income level

B. Help individuals better integrate into society

3. If you're watching TV at home on a Sunday morning, there are two documentaries you can choose to watch:

A. Nature: Our Earth B. Sex and Love

4. Which of the following questions would you like to discuss with others?

A. Movies from the '90s

B. World natural heritage

5. If you had the necessary skills, which of the following positions would you like to have:

A. Superintendent of schools for the blind

B. Responsible person of environmental materials company

Hedonism：

1. If you're watching TV at home on a Sunday morning, there are two documentaries you can choose to watch:

A. Nature: Our Earth B. The secrets of happiness

2. If you go to an exhibition, you tend to go to:

A. The latest electronic goods, such as home appliances and cars

B. Daily needs, such as snacks, wine ects.

3. You are arranged to wait in the lounge. There are two magazines to distract you. You will choose:

A. Art and decoration

B. Travel and food

4. When you choose a private travel destination, you will consider:

A. cities with large shopping malls

B. a city with many museums and libraries

5. If you had a day off, what would you choose?

A. Eat and drink

B. Exercise with friends

A balanced diet is very important for human health. Excessive consumption of sugar, oil and fat can lead to a high risk of the disease. In a study published in 2014 in *JAMA Internal Medicine*, Dr. Hu and his colleagues found an association between a high-sugar diet and a greater risk of dying from heart disease. Over the course of the 15-year study, people between 16-30 years old who got 17% to 21% of their calories from added sugar had a 38% higher risk of dying from cardiovascular disease compared with those who consumed 8% of their calories as added sugar. Besides, another study published in 2019 also found the overconsumption of oil and fat will lead to high cholesterol levels, which is also the leading cause of the serious cardiovascular disease between young people.

1. To what extent do you feel uncomfortable when reading the message?(1=not at all, 7= very uncomfortable)

2. To what extent do you feel stressful when reading the message?(1=not at all, 7= very stressful)

3. To what extent do you feel sorry for what the message described? (1=not at all, 7= very sorry)

Can you please write twenty two in Arabic numbers below (attention check)

In the next step, we will provide you several food options, you can choose one you are most preferred to buy in each round.

4. Which drink you would like to buy?

<table>
<tr>
<td>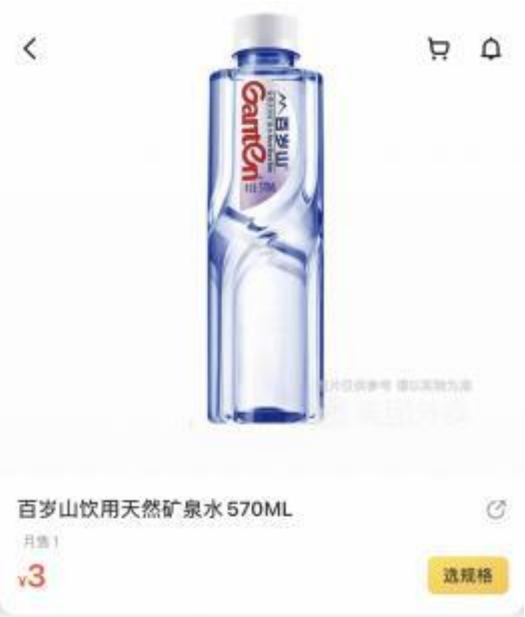
</td>
<td>Name: Mineral water
570 ml
Calories: 0 kcal/100 ml</td>
</tr>
</table>

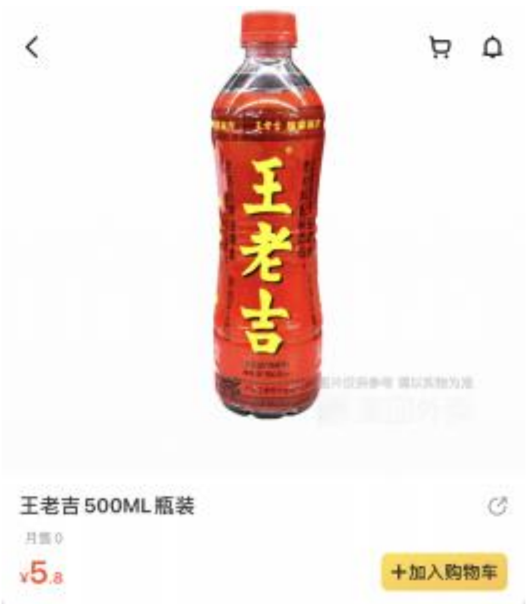	Name: Wanglaoji herbal tea<br>500 ml<br>Calories: 37.35 kcal/100 ml<br>Sugar：8 g/100 ml
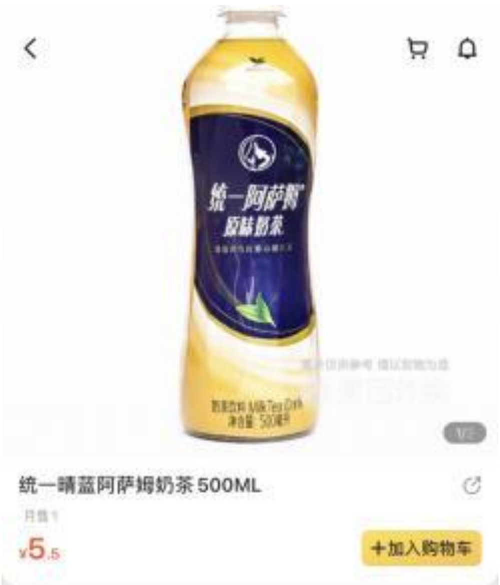	Name: Tongyi Milk tea<br>500 ml<br>Calories：55 kcal/100 ml<br>Sugar：10 g/100 ml<br>Protein：1.7 g/100 ml<br>Carbohydrate：9.2 g/100 ml
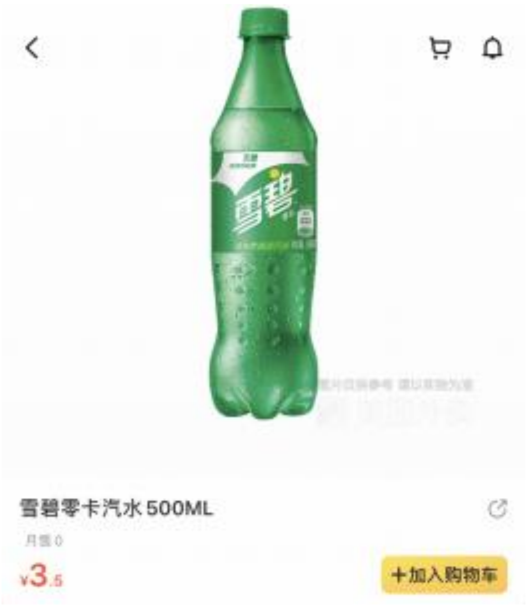	Name: Sprite (no sugar)<br>500 ml<br>Calories：0 kcal<br>Dietary fiber：1.5 g/100 ml

5. Which meat you would like to buy?

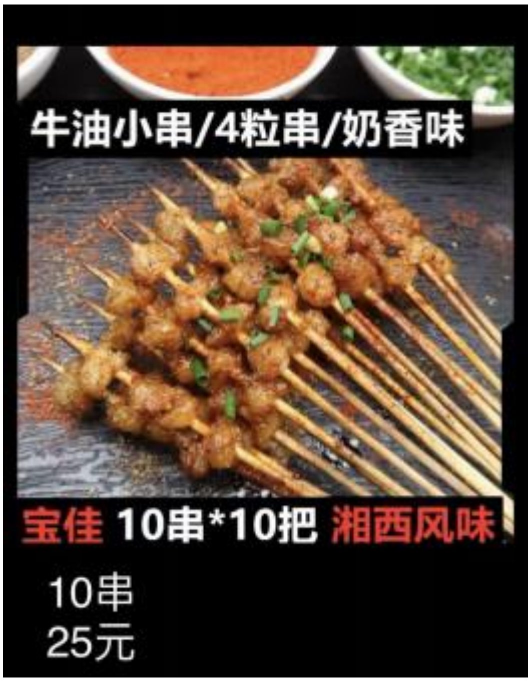	Name：Roast beef marrow<br>Calories： 430 kcal/100 g<br>Sugar：5 g/100 g<br>Fat：25.2 g/100 g<br>Carbohydrate：26.9 g/100 g
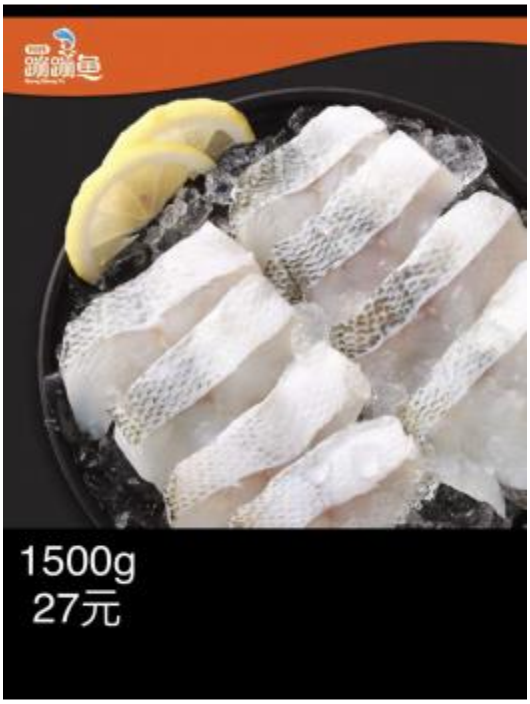	Name: sashimi<br>Calories：100 kcal/100 g<br>Protein：23.2 g/100 g<br>Fat：0.1 g/100 g

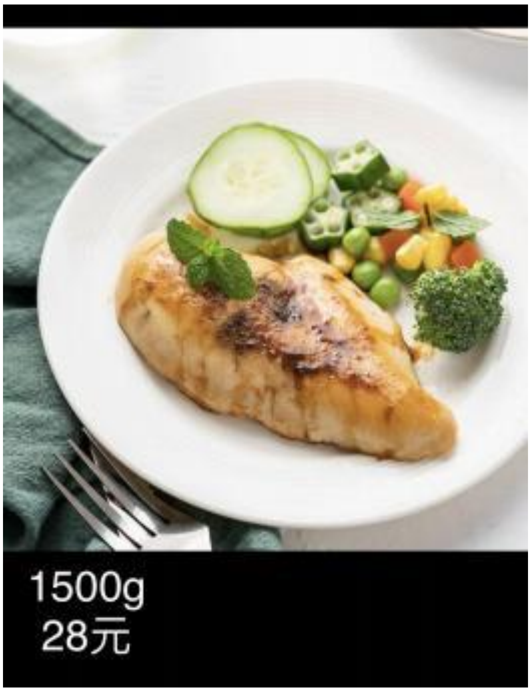	Name：fry chicken breast<br>Calories：110 kcal/100 g<br>Protein：27.6 g/100 g<br>Fat：1.6 g/100 g
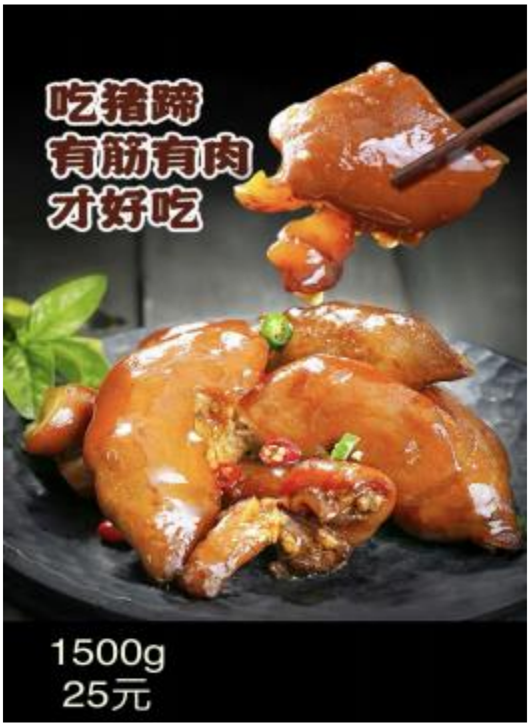	Name: simmered pig feet<br>Calories：237 kcal/100 g<br>Protein：28.4 g/100 g<br>Fat：15.4 g/100 g

Demographics

1. What's your age?
2. What's your gender?
3. What is your highest level of education so far?

o Bachelor
o Master
o PhD or above

4. What's your monthly income (including contribution and scholarship) ? (RMB)
o Lower than 1000
o 1000 ~ 1500
o 1500 ~ 2000
o 2000 ~ 2500
o 2500 ~ 3000
o Above 3000

Thank you for participating in our survey. Your answer has been recorded.It is very much appreciated.
If you have any questions regarding the experiments or if you would like to receive the results of the survey you can contact us by j.li.32@student.rug.nl.
This is the end of the survey, thank you!

Treatment group 1 (Negative self-evaluative emotions manipulated)

1. What aspects of your life are medium important to you?

There are always some aspects of our lives that are important to us and some aspects that are less important. Based on your own experience, choose the one aspect of your life that you think is medium important to you.

o Theory: Science, technology and research progress are important to me
o Economics: Practicality, money and things related to finance are important to me
o Aesthetics: Art, music and other art-related things are very important to me
o Social aspects: People, society, relationships and social order are important to me
o Political aspects: Leadership, domination and authority are important to me
o Religion: God, faith and religious practices are important to me
o Environment: Nature, the earth, animals and other species are important to me
o Hedonic: It's important for me to enjoy life and satisfy my desires

Theory:

6. If you are watching TV, there are two documentaries for you to choose from, which you will watch:

A. space exploration B. overseas investment

7. Which of the following do you think has made the most contribution to the progress of human society

A. Newton, the founder of theoretical physics

B. Martin Luther King,the champion of human rights

8. Which of the following areas do you think is more important for human beings:

A. mathematics and physics B. theology

9. If you are a professor and you have the necessary knowledge, you would like to teach:

A. literature and art B. physics and chemistry

10. If you see two articles in a newspaper of the same length, which one do you choose to read?

A. The political summit will beheld tomorrow   B. Major scientific discovery

Economy：

6. In your opinion, the main influence of the European Union is:
A. facilitated free international trade
B. formed a political bloc with strong influence in international affairs
7. Which of the following functions do you think is the most important for a modern leader:
A. Ensuring that goals are achieved
B. Encouraging mutual respect among subordinates
8. If you saw two articles in a newspaper of the same length, which one would you choose to read?
A. Leaders of religious groups work together to promote unity
B. the international market environment has greatly improved
9. If you go to an exhibition, you tend to go to:
A. The latest electronic goods, such as home appliances and cars
B. Scientific research equipment, such as medical equipment
10. Which role of education do you think is more important:
A. Provide individuals with the competitiveness to increase their income level
B. Help individuals better integrate into society

Aethetics：

6. Do you accept that great artists such as Mozart, Shakespeare, and Picasso were selfish or even harmful:
A. Yes   B. No
7. Which of the following questions would you like to discuss with others?
A. Movies from the '90s
B. European relations after the fall of the Berlin Wall
8. You are sick in bed on Sunday, it is you hear a radio program, you wish it was:
A. Pop music   B. Religious programs
9. If you were given the chance to form a group of your own, what would you choose?
A. Political groups
B. Bands or other art groups
10. Current technological developments mean that we have reached a stage of social civilization that is more advanced than earlier civilizations such as ancient Greece:
A. Yes   B. No

Social aspects：

6. Which of the following do you think has made the most contribution to the progress of human society:
A. Newton, the founder of theoretical physics   B. Martin Luther
7. Do you accept that great artists such as Mozart, Shakespeare, and Picasso were selfish or even harmful:
A. Yes   B. No
8. Which of the following functions do you think is the most important for a modern leader:
A. Ensuring that goals are achieved   B. Encouraging mutual respect among subordinates
9. Would you like to donate to:
A. Missionary organizations   B. Disaster relief groups
10. Which role of education do you think is more important:

A. Provide individuals with the competitiveness to increase their income level
B. Help individuals better integrate into society

Political aspects：
6. In your opinion, the main influence of the European Union is:
A. facilitated free international trade
B. formed a political bloc with strong influence in international affairs
7. Which of the following questions would you like to discuss with others?
A. Movies from the '90s
B. European relations after the fall of the Berlin Wall
8. If you were given the chance to form a group of your own, what would you choose?
A. Political groups
B. Bands or other art groups
9. If you see two articles in a newspaper of the same length, which one do you choose to read?
A. The political summit will beheld tomorrow B. Major scientific discovery
10. Which of the following do you think deserves better recognition:
A. a world-class politician B. a Nobel Prize winner for literature

Religion：
6. The Bible, as a book, has great literary value over its religious value:
A. Yes B. No
7. Which of the following areas do you think is more important for human beings:
A. mathematics and physics B. theology
8. Which organization would you rather donate to:
A. religious communication working group B. refugee relief group
9. If you saw two articles in a newspaper of the same length, which one would you choose to read?
A. Leaders of religious groups work together to promote unity
B. the international market environment has greatly improved
10. You are sick in bed on Sunday, it is you hear a radio program, you wish it was:
A. Pop music B. Religious programs

Environments：
6. Which organization would you rather donate to:
A. The Society for the Protection of Animals
B. The Society for Disease Prevention
7. Which role of education do you think is more important:
A. Provide individuals with the competitiveness to increase their income level
B. Help individuals better integrate into society
8. If you're watching TV at home on a Sunday morning, there are two documentaries you can choose to watch:
A. Nature: Our Earth B. Sex and Love
9. Which of the following questions would you like to discuss with others?
A. Movies from the '90s
B. World natural heritage
10. If you had the necessary skills, which of the following positions would you like to have:
A. Superintendent of schools for the blind

B. Responsible person of environmental materials company

Hedonism：

6. If you're watching TV at home on a Sunday morning, there are two documentaries you can choose to watch:
A. Nature: Our Earth B. The secrets of happiness
7. If you go to an exhibition, you tend to go to:
A. The latest electronic goods, such as home appliances and cars
B. Daily needs, such as snacks, wine ects.
8. You are arranged to wait in the lounge. There are two magazines to distract you. You will choose:
A. Art and decoration
B. Travel and food
9. When you choose a private travel destination, you will consider:
A. cities with large shopping malls
B. a city with many museums and libraries
10. If you had a day off, what would you choose?
A. Eat and drink
B. Exercise with friends

A balanced diet is very important for human health. Excessive consumption of sugar, oil and fat can lead to a high risk of the disease. In a study published in 2014 in *JAMA Internal Medicine*, Dr. Hu and his colleagues found an association between a high-sugar diet and a greater risk of dying from heart disease. Over the course of the 15-year study, people between 16-30 years old who got 17% to 21% of their calories from added sugar had a 38% higher risk of dying from cardiovascular disease compared with those who consumed 8% of their calories as added sugar. Besides, another study published in 2019 also found the overconsumption of oil and fat will lead to high cholesterol levels, which is also the leading cause of the serious cardiovascular disease between young people.

Yu Hailong, a well-known food-sharing blogger, died on March 5 at the age of 29, according to media reports. According to medical experts, Yu Hailong's sudden death was caused by long-term overeating and staying up late. It is reported that Yu Hailong has long been suffering from chronic diseases due to obesity, but due to the nature of its work (food sharing), it cannot be alleviated and treated.

2. To what extent do you feel uncomfortable when reading the message? (1=not at all, 7= very uncomfortable)

3. To what extent do you feel stressful when reading the message? (1=not at all, 7= very stressful)

4. To what extent do you feel sorry for what the message described? (1=not at all, 7= very sorry)

Can you please write twenty two in Arabic numbers blow:____________

In the next step, we will provide you several food options, you can choose one you are most preferred to buy in each round.

5. Which drink you would like to buy?

<table>
<tr>
<td>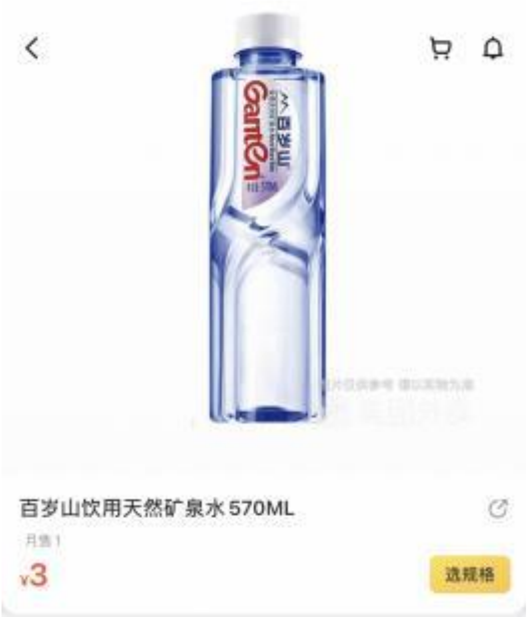
</td>
<td>Name: Mineral water
570 ml
Calories：0kcal/100ml</td>
</tr>
<tr>
<td>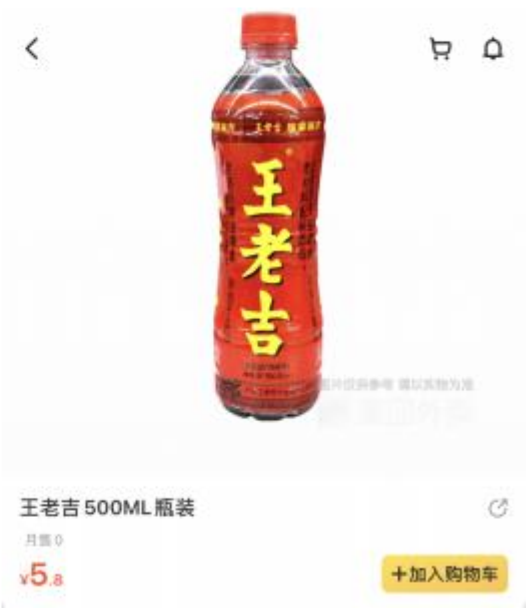
</td>
<td>Name: Wanglaoji herbal tea
500ml
Calories：37.35 kcal /100ml
Sugar：8g / 100ml</td>
</tr>
</table>

<table>
<tr>
<td>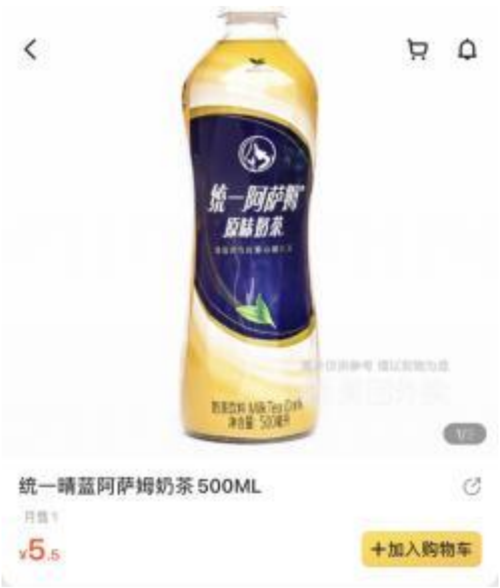
</td>
<td>Name: Tongyi Milk tea
500ml
Calories：55kcal/100ml
Sugar：10g / 100ml
Protein：1.7g / 100ml
Carbohydrate：9.2g / 100ml</td>
</tr>
<tr>
<td>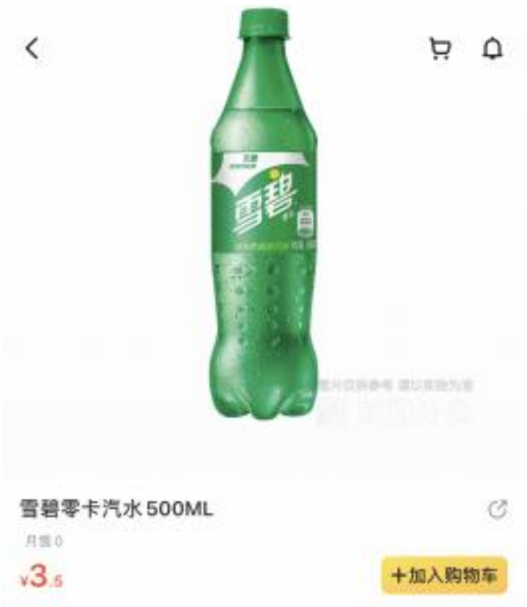
</td>
<td>Name: Sprite (no sugar)
500ml
Calories：0 kcal
Dietary fiber：1.5g/100ml</td>
</tr>
</table>

6. Which meat you would like to buy?

<table>
<tr>
<td>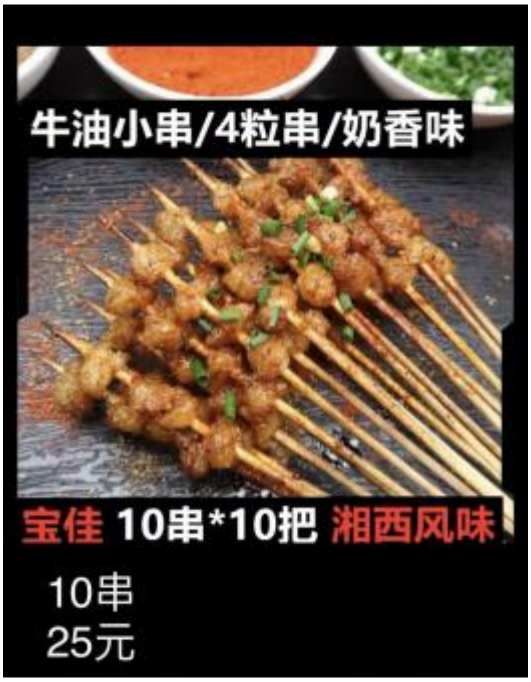
</td>
<td>Name：Roast beef marrow
Calories： 430kcal/100g
Sugar：5g/100g
Fat：25.2g/100g
Carbohydrate：26.9g/100g</td>
</tr>
</table>

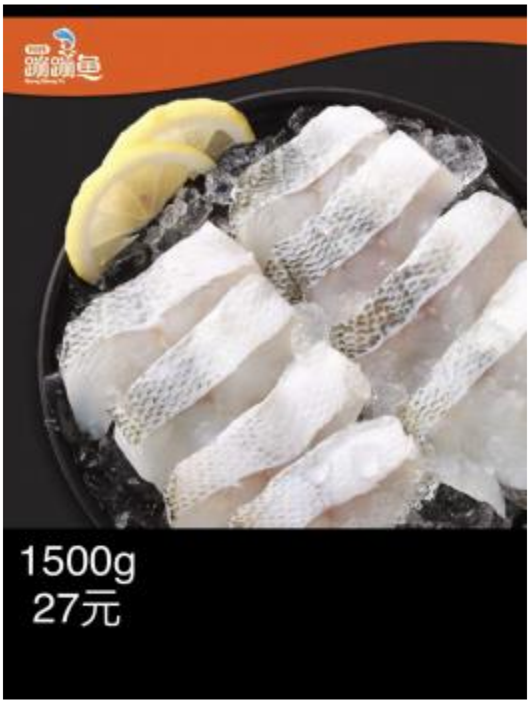	Name: sashimi<br>Calories：100kcal/100g<br>Protein：23.2g/100g<br>Fat：0.1g/100g
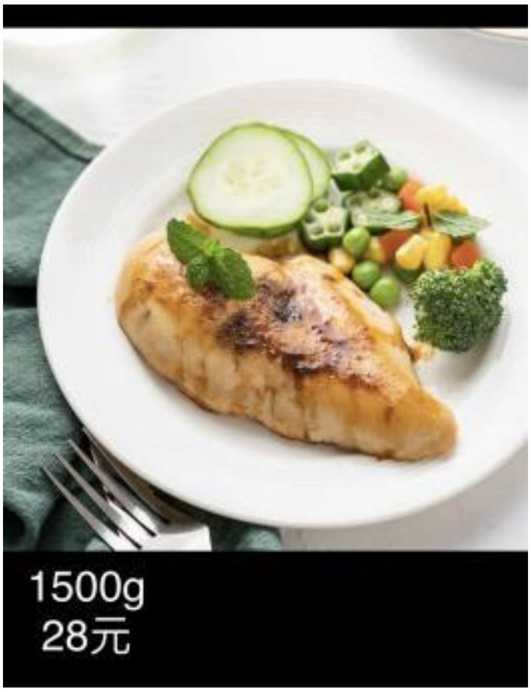	Name：fry chicken breast<br>Calories：110kcal/100g<br>Protein：27.6g/100g<br>Fat：1.6g/100g

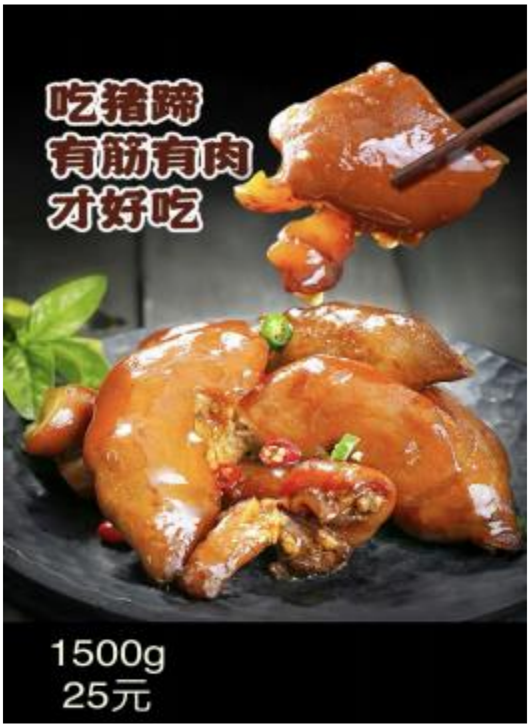	Name: simmered pig feet<br>Calories：237kcal/100g<br>Protein：28.4g/100g<br>Fat：15.4g/100g

Demographics

5. What's your age?
6. What's your gender?
7. What is your highest level of education so far?

o Bachelor
o Master
o PhD or above

8. What's your monthly income (including contribution and scholarship) ? (RMB)

o Lower than 1000
o 1000 ~ 1500
o 1500 ~ 2000
o 2000 ~ 2500
o 2500 ~ 3000
o Above 3000

Thank you for participating in our survey. Your answer has been recorded.It is very much appreciated.
If you have any questions regarding the experiments or if you would like to receive the results of the survey you can contact us by j.li.32@student.rug.nl.
This is the end of the survey, thank you!

Treatment group 2 (self-affirmation manipulated)

1. What aspects of your life are most important to you?

There are always some aspects of our lives that are important to us and some aspects that are less important. Based on your own experience, choose the one aspect of your life that you think is most important to you.

o Theory: Science, technology and research progress are important to me

o Economics: Practicality, money and things related to finance are important to me
o Aesthetics: Art, music and other art-related things are very important to me
o Social life: People, society, relationships and social order are important to me
o Politics: Leadership, domination and authority are important to me
o Religion: God, faith and religious practices are important to me
o Environment: Nature, the earth, animals and other species are important to me
o Hedonic: It's important for me to enjoy life and satisfy my desires

Theory:
11. If you are watching TV, there are two documentaries for you to choose from, which you will watch:
A. space exploration B. overseas investment
12. Which of the following do you think has made the most contribution to the progress of human society:
A. Newton, the founder of theoretical physics
B. Martin Luther King,the champion of human rights
13. Which of the following areas do you think is more important for human beings:
A. mathematics and physics
B. theology
14. If you are a professor and you have the necessary knowledge, you would like to teach:
A. literature and art
B. physics and chemistry
15. If you see two articles in a newspaper of the same length, which one do you choose to read?
A. The political summit will beheld tomorrow B. Major scientific discovery

Economy：
11. In your opinion, the main influence of the European Union is:
A. facilitated free international trade
B. formed a political bloc with strong influence in international affairs
12. Which of the following functions do you think is the most important for a modern leader:
A. Ensuring that goals are achieved
B. Encouraging mutual respect among subordinates
13. If you saw two articles in a newspaper of the same length, which one would you choose to read?
A. Leaders of religious groups work together to promote unity
B. the international market environment has greatly improved
14. If you go to an exhibition, you tend to go to:
A. The latest electronic goods, such as home appliances and cars
B. Scientific research equipment, such as medical equipment
15. Which role of education do you think is more important:
A. Provide individuals with the competitiveness to increase their income level
B. Help individuals better integrate into society

Aethetics：
11. Do you accept that great artists such as Mozart, Shakespeare, and Picasso were selfish or even harmful:

A. Yes B. No
12. Which of the following questions would you like to discuss with others?
A. Movies from the '90s
B. European relations after the fall of the Berlin Wall
13. You are sick in bed on Sunday, it is you hear a radio program, you wish it was:
A. Pop music B. Religious programs
14. If you were given the chance to form a group of your own, what would you choose?
A. Political groups
B. Bands or other art groups
15. Current technological developments mean that we have reached a stage of social civilization that is more advanced than earlier civilizations such as ancient Greece:
A. Yes B. No

Social aspects：
11. Which of the following do you think has made the most contribution to the progress of human society:
A. Newton, the founder of theoretical physics B. Martin Luther
12. Do you accept that great artists such as Mozart, Shakespeare, and Picasso were selfish or even harmful:
A. Yes B. No
13. Which of the following functions do you think is the most important for a modern leader:
A. Ensuring that goals are achieved
B. Encouraging mutual respect among subordinates
14. Would you like to donate to:
A. Missionary organizations B. Disaster relief groups
15. Which role of education do you think is more important:
A. Provide individuals with the competitiveness to increase their income level
B. Help individuals better integrate into society

Political aspects：
11. In your opinion, the main influence of the European Union is:
A. facilitated free international trade
B. formed a political bloc with strong influence in international affairs
12. Which of the following questions would you like to discuss with others?
A. Movies from the '90s
B. European relations after the fall of the Berlin Wall
13. If you were given the chance to form a group of your own, what would you choose?
A. Political groups
B. Bands or other art groups
14. If you see two articles in a newspaper of the same length, which one do you choose to read?
A. The political summit will beheld tomorrow
B. Major scientific discovery
15. Which of the following do you think deserves better recognition:
A. a world-class politician B. a Nobel Prize winner for literature

Religion：
11. The Bible, as a book, has great literary value over its religious value:

A. Yes B. No

12. Which of the following areas do you think is more important for human beings:

A. mathematics and physics B. theology

13. Which organization would you rather donate to:

A. religious communication working group B. refugee relief group

14. If you saw two articles in a newspaper of the same length, which one would you choose to read?

A. Leaders of religious groups work together to promote unity

B. the international market environment has greatly improved

15. You are sick in bed on Sunday, it is you hear a radio program, you wish it was:

A. Pop music B. Religious programs

Environments：

11. Which organization would you rather donate to:

A. The Society for the Protection of Animals

B. The Society for Disease Prevention

12. Which role of education do you think is more important:

A. Provide individuals with the competitiveness to increase their income level

B. Help individuals better integrate into society

13. If you're watching TV at home on a Sunday morning, there are two documentaries you can choose to watch:

A. Nature: Our Earth

B. Sex and Love

14. Which of the following questions would you like to discuss with others?

A. Movies from the '90s

B. World natural heritage

15. If you had the necessary skills, which of the following positions would you like to have:

A. Superintendent of schools for the blind

B. Responsible person of environmental materials company

Hedonism：

11. If you're watching TV at home on a Sunday morning, there are two documentaries you can choose to watch:

A. Nature: Our Earth B. The secrets of happiness

12. If you go to an exhibition, you tend to go to:

A. The latest electronic goods, such as home appliances and cars

B. Daily needs, such as snacks, wine ects.

13. You are arranged to wait in the lounge. There are two magazines to distract you. You will choose:

A. Art and decoration

B. Travel and food

14. When you choose a private travel destination, you will consider:

A. cities with large shopping malls

B. a city with many museums and libraries

15. If you had a day off, what would you choose?

A. Eat and drink

B. Exercise with friends

A balanced diet is very important for human health. Excessive consumption of sugar, oil and fat can lead to a high risk of the disease. In a study published in 2014 in *JAMA Internal Medicine*, Dr. Hu and his colleagues found an association between a high-sugar diet and a greater risk of dying from heart disease. Over the course of the 15-year study, people between 16-30 years old who got 17% to 21% of their calories from added sugar had a 38% higher risk of dying from cardiovascular disease compared with those who consumed 8% of their calories as added sugar. Besides, another study published in 2019 also found the overconsumption of oil and fat will lead to high cholesterol levels, which is also the leading cause of the serious cardiovascular disease between young people.

2. To what extent do you feel uncomfortable when reading the message?(1=not at all, 7= very uncomfortable)

3. To what extent do you feel stressful when reading the message?(1=not at all, 7= very stressful)

4. To what extent do you feel sorry for what the message described? (1=not at all, 7= very sorry)

Can you please write twenty two in Arabic numbers blow:

____

In the next step, we will provide you several food options, you can choose one you are most preferred to buy in each round.

5. Which drink you would like to buy?

<table>
<tr>
<td>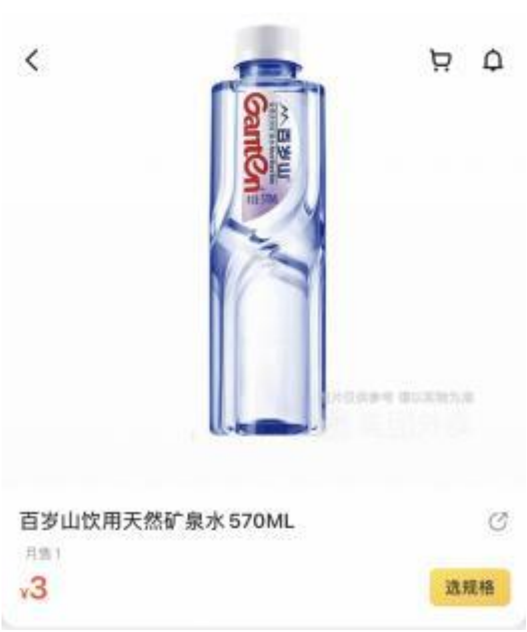
</td>
<td>Name: Mineral water
570 ml
Calories：0kcal/100ml</td>
</tr>
</table>

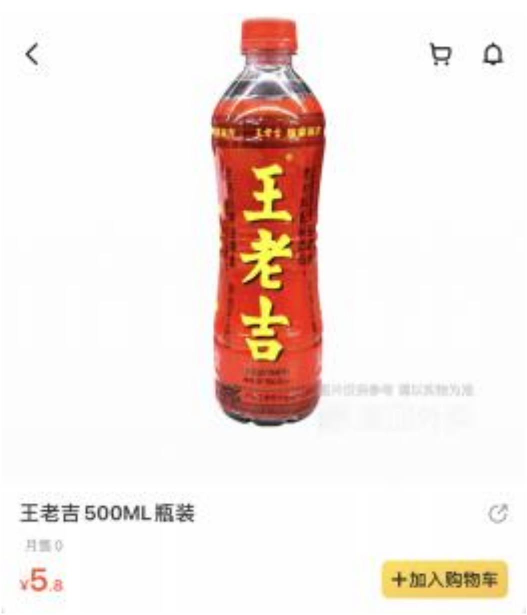	Name: Wanglaoji herbal tea<br>500ml<br>Calories：37.35 kcal /100ml<br>Sugar：8g / 100ml
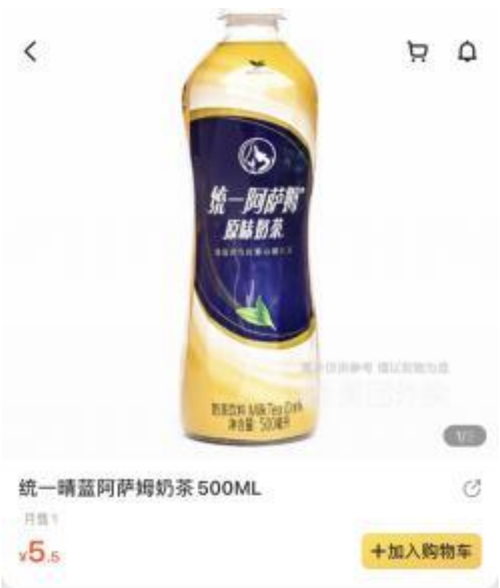	Name: Tongyi Milk tea<br>500ml<br>Calories：55kcal/100ml<br>Sugar：10g / 100ml<br>Protein：1.7g / 100ml<br>Carbohydrate：9.2g / 100ml
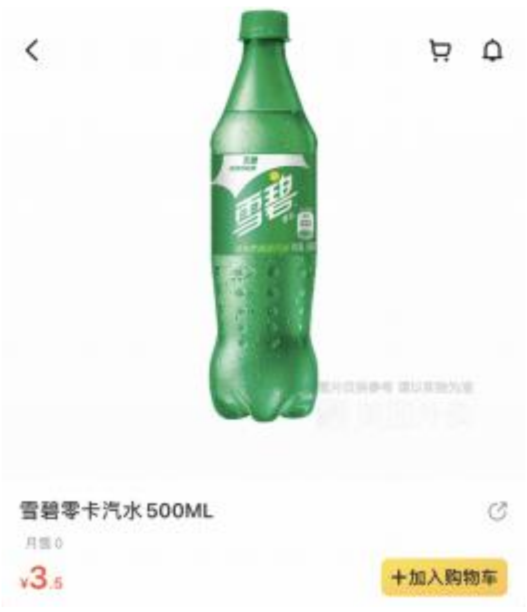	Name: Sprite (no sugar)<br>500ml<br>Calories：0 kcal<br>Dietary fiber：1.5g/100ml

6. Which meat you would like to buy?

<table>
<tr><td>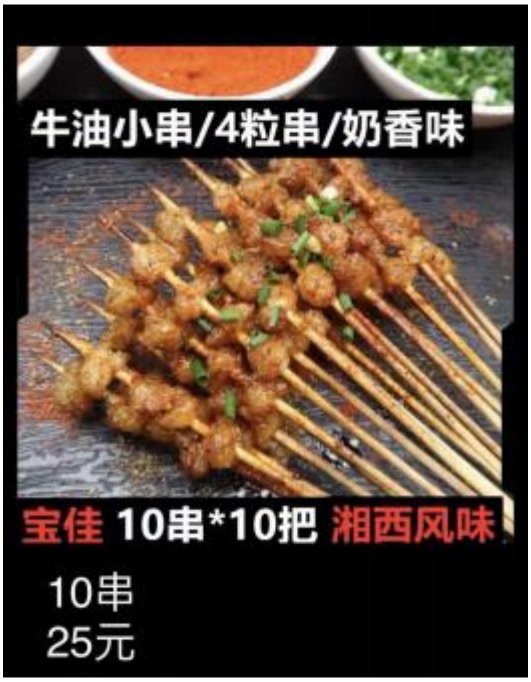
</td><td>Name：Roast beef marrow
Calories： 430kcal/100g
Sugar：5g/100g
Fat：25.2g/100g
Carbohydrate：26.9g/100g</td></tr>
<tr><td>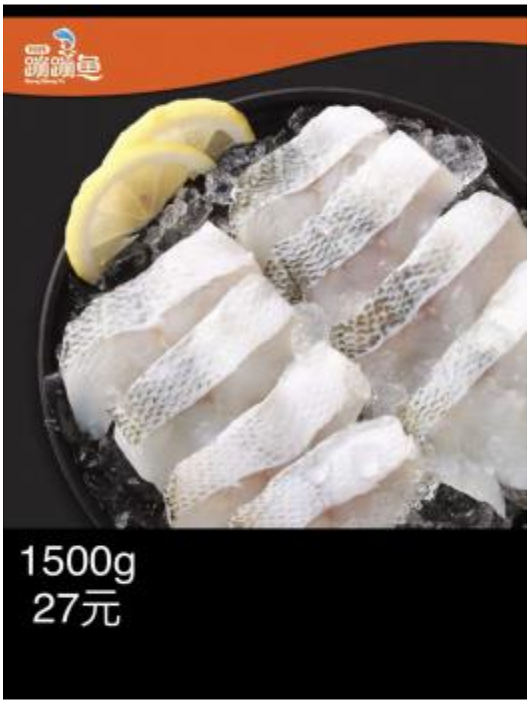
</td><td>Name: sashimi
Calories：100kcal/100g
Protein：23.2g/100g
Fat：0.1g/100g</td></tr>
</table>

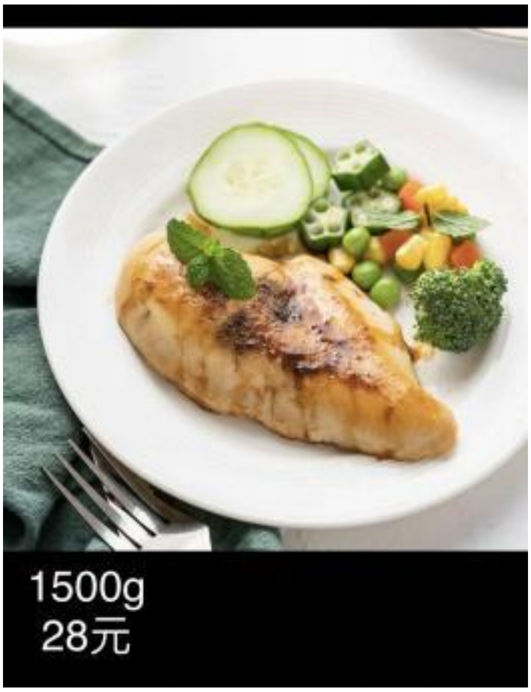	Name：fry chiken breast<br>Calories：110kcal/100g<br>Protein：27.6g/100g<br>Fat：1.6g/100g
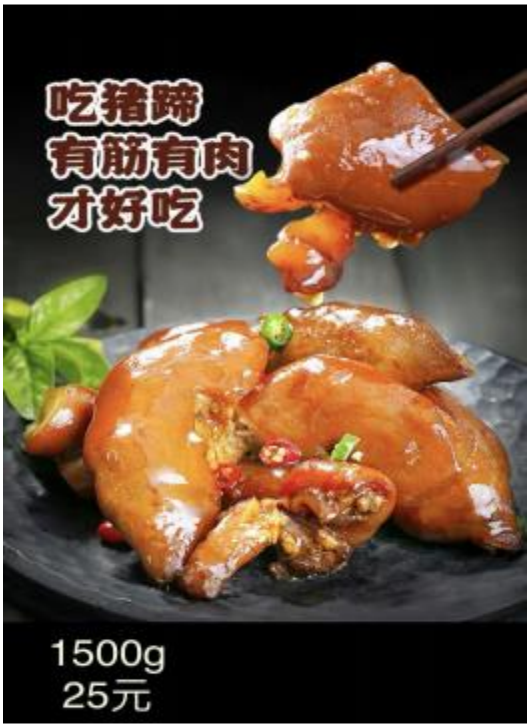	Name: simmered pig feet<br>Calories：237kcal/100g<br>Protein：28.4g/100g<br>Fat：15.4g/100g

Demographics

9. What's your age?
10. What's your gender?
11. What is your highest level of education so far?

o Bachelor

o Master
o PhD or above
12. What’s your monthly income (including contribution and scholarship) ? (RMB)
o Lower than 1000
o 1000 ~ 1500
o 1500 ~ 2000
o 2000 ~ 2500
o 2500 ~ 3000
o Above 3000

Thank you for participating in our survey. Your answer has been recorded.It is very much appreciated.
If you have any questions regarding the experiments or if you would like to receive the results of the survey you can contact us by j.li.32@student.rug.nl.
This is the end of the survey, thank you!

Treatment group 3 (Negative self-evaluative emotions and self-affirmation both manipulated)

1. What aspects of your life are most important to you?

There are always some aspects of our lives that are important to us and some aspects that are less important. Based on your own experience, choose the one aspect of your life that you think is most important to you.

o Theory: Science, technology and research progress are important to me
o Economics: Practicality, money and things related to finance are important to me
o Aesthetics: Art, music and other art-related things are very important to me
o Social life: People, society, relationships and social order are important to me
o Politics: Leadership, domination and authority are important to me
o Religion: God, faith and religious practices are important to me
o Environment: Nature, the earth, animals and other species are important to me
o Hedonic: It's important for me to enjoy life and satisfy my desires

Theory:
16. If you are watching TV, there are two documentaries for you to choose from, which you will watch:
A. space exploration B. overseas investment
17. Which of the following do you think has made the most contribution to the progress of human society:
A. Newton, the founder of theoretical physics B. Martin Luther King,the champion of human rights
18. Which of the following areas do you think is more important for human beings:
A. mathematics and physics B. theology
19. If you are a professor and you have the necessary knowledge, you would like to teach:
A. literature and art B. physics and chemistry
20. If you see two articles in a newspaper of the same length, which one do you choose to read?

A. The political summit will beheld tomorrow B. Major scientific discovery

Economy：
16. In your opinion, the main influence of the European Union is:
A. facilitated free international trade
B. formed a political bloc with strong influence in international affairs
17. Which of the following functions do you think is the most important for a modern leader:
A. Ensuring that goals are achieved
B. Encouraging mutual respect among subordinates
18. If you saw two articles in a newspaper of the same length, which one would you choose to read?
A. Leaders of religious groups work together to promote unity
B. the international market environment has greatly improved
19. If you go to an exhibition, you tend to go to:
A. The latest electronic goods, such as home appliances and cars
B. Scientific research equipment, such as medical equipment
20. Which role of education do you think is more important:
A. Provide individuals with the competitiveness to increase their income level
B. Help individuals better integrate into society

Aethetics：
16. Do you accept that great artists such as Mozart, Shakespeare, and Picasso were selfish or even harmful:
A. Yes B. No
17. Which of the following questions would you like to discuss with others?
A. Movies from the '90s
B. European relations after the fall of the Berlin Wall
18. You are sick in bed on Sunday, it is you hear a radio program, you wish it was:
A. Pop music B. Religious programs
19. If you were given the chance to form a group of your own, what would you choose?
A. Political groups
B. Bands or other art groups
20. Current technological developments mean that we have reached a stage of social civilization that is more advanced than earlier civilizations such as ancient Greece:
A. Yes B. No

Social aspects：
16. Which of the following do you think has made the most contribution to the progress of human society:
A. Newton, the founder of theoretical physics
B. Martin Luther
17. Do you accept that great artists such as Mozart, Shakespeare, and Picasso were selfish or even harmful:
A. Yes B. No
18. Which of the following functions do you think is the most important for a modern leader:
A. Ensuring that goals are achieved
B. Encouraging mutual respect among subordinates
19. Would you like to donate to:

A. Missionary organizations
B. Disaster relief groups
20. Which role of education do you think is more important:
A. Provide individuals with the competitiveness to increase their income level
B. Help individuals better integrate into society

Political aspects：
16. In your opinion, the main influence of the European Union is:
A. facilitated free international trade
B. formed a political bloc with strong influence in international affairs
17. Which of the following questions would you like to discuss with others?
A. Movies from the '90s
B. European relations after the fall of the Berlin Wall
18. If you were given the chance to form a group of your own, what would you choose?
A. Political groups
B. Bands or other art groups
19. If you see two articles in a newspaper of the same length, which one do you choose to read?
A. The political summit will beheld tomorrow B. Major scientific discovery
20. Which of the following do you think deserves better recognition:
A. a world-class politician
B. a Nobel Prize winner for literature

Religion：
16. The Bible, as a book, has great literary value over its religious value:
A. Yes B. No
17. Which of the following areas do you think is more important for human beings:
A. mathematics and physics B. theology
18. Which organization would you rather donate to:
A. religious communication working group B. refugee relief group
19. If you saw two articles in a newspaper of the same length, which one would you choose to read?
A. Leaders of religious groups work together to promote unity
B. the international market environment has greatly improved
20. You are sick in bed on Sunday, it is you hear a radio program, you wish it was:
A. Pop music B. Religious programs

Environments：
16. Which organization would you rather donate to:
A. The Society for the Protection of Animals
B. The Society for Disease Prevention
17. Which role of education do you think is more important:
A. Provide individuals with the competitiveness to increase their income level
B. Help individuals better integrate into society
18. If you're watching TV at home on a Sunday morning, there are two documentaries you can choose to watch:
A. Nature: Our Earth B. Sex and Love
19. Which of the following questions would you like to discuss with others?
A. Movies from the '90s

B. World natural heritage
20. If you had the necessary skills, which of the following positions would you like to have:
A. Superintendent of schools for the blind
B. Responsible person of environmental materials company

Hedonism：
16. If you're watching TV at home on a Sunday morning, there are two documentaries you can choose to watch:
A. Nature: Our Earth B. The secrets of happiness
17. If you go to an exhibition, you tend to go to:
A. The latest electronic goods, such as home appliances and cars
B. Daily needs, such as snacks, wine ects.
18. You are arranged to wait in the lounge. There are two magazines to distract you. You will choose:
A. Art and decoration
B. Travel and food
19. When you choose a private travel destination, you will consider:
A. cities with large shopping malls
B. a city with many museums and libraries
20. If you had a day off, what would you choose?
A. Eat and drink
B. Exercise with friends

A balanced diet is very important for human health. Excessive consumption of sugar, oil and fat can lead to a high risk of the disease. In a study published in 2014 in *JAMA Internal Medicine*, Dr. Hu and his colleagues found an association between a high-sugar diet and a greater risk of dying from heart disease. Over the course of the 15-year study, people between 16-30 years old who got 17% to 21% of their calories from added sugar had a 38% higher risk of dying from cardiovascular disease compared with those who consumed 8% of their calories as added sugar. Besides, another study published in 2019 also found the overconsumption of oil and fat will lead to high cholesterol levels, which is also the leading cause of the serious cardiovascular disease between young people.

Yu Hailong, a well-known food-sharing blogger, died on March 5 at the age of 29, according to media reports. According to medical experts, Yu Hailong's sudden death was caused by long-term overeating and staying up late. It is reported that Yu Hailong has long been suffering from chronic diseases due to obesity, but due to the nature of its work (food sharing), it cannot be alleviated and treated.

2. To what extent do you feel uncomfortable when reading the message?(1=not at all, 7= very uncomfortable)

3. To what extent do you feel stressful when reading the message?(1=not at all, 7= very stressful)

4. To what extent do you feel sorry for what the message described? (1=not at all, 7= very sorry)

Can you write twenty two in Arabic numbers below:

____

In the next step, we will provide you several food options, you can choose one you are most preferred to buy in each round.

5. Which drink you would like to buy?

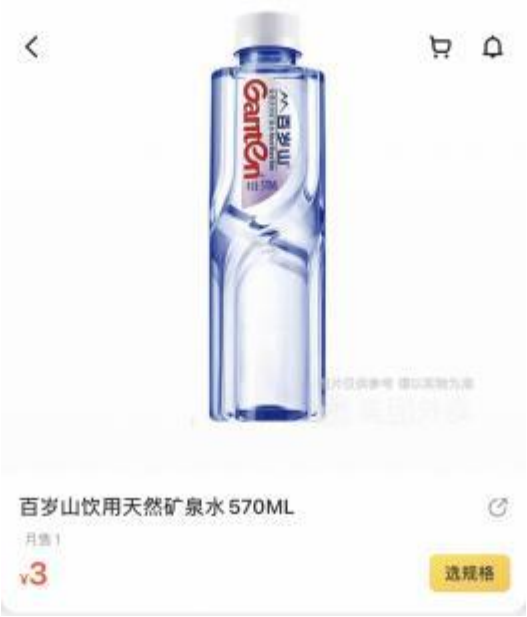	Name: Mineral water<br>570 ml<br>Calories：0kcal/100ml
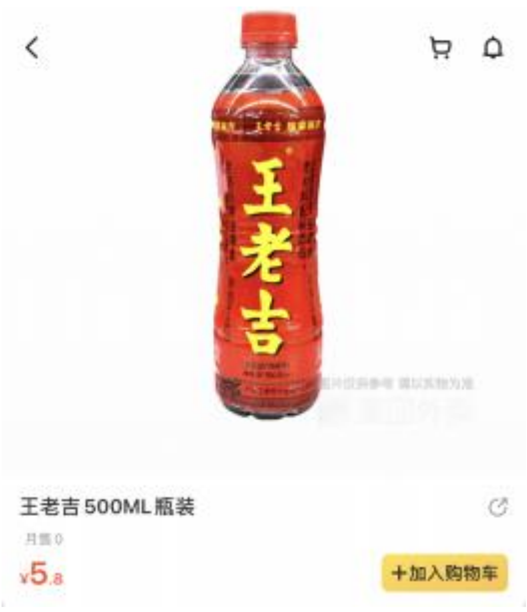	Name: Wanglaoji herbal tea<br>500ml<br>Calories：37.35 kcal /100ml<br>Sugar：8g / 100ml

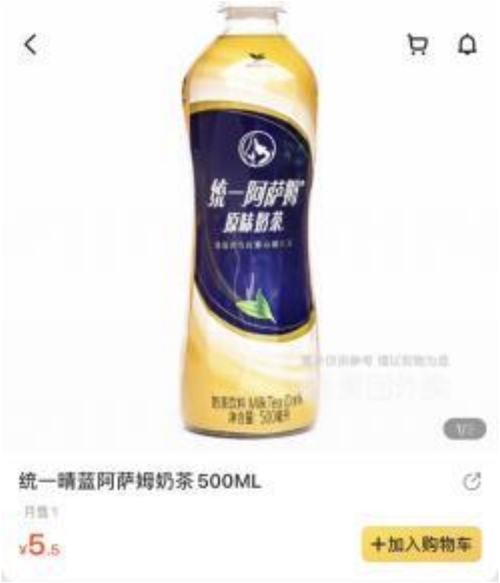	Name: Tongyi Milk tea<br>500ml<br>Calories：55kcal/100ml<br>Sugar：10g / 100ml<br>Protein：1.7g / 100ml<br>Carbohydrate：9.2g / 100ml
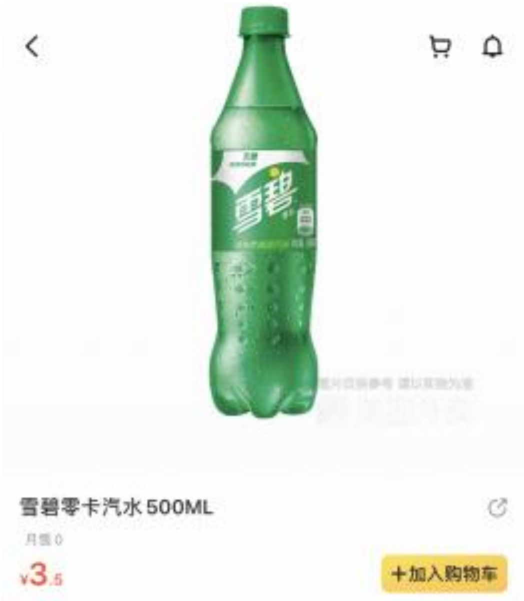	Name: Sprite (no sugar)<br>500ml<br>Calories：0 kcal<br>Dietary fiber：1.5g/100ml

6. Which meat you would like to buy?

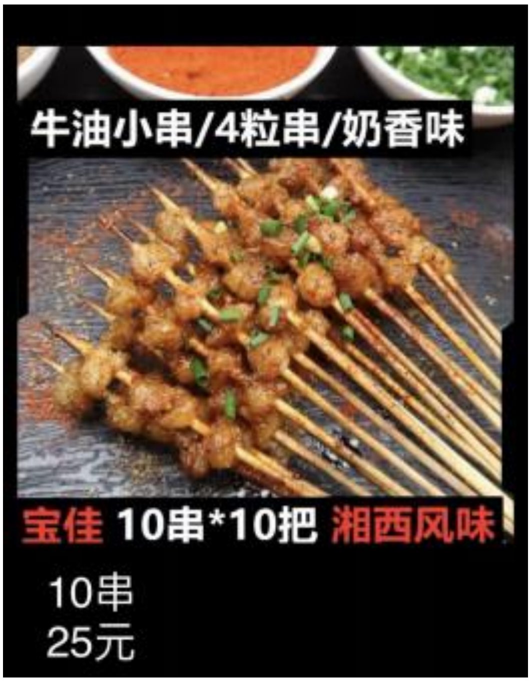	Name：Roast beef marrow<br>Calories： 430kcal/100g<br>Sugar：5g/100g<br>Fat：25.2g/100g<br>Carbohydrate：26.9g/100g
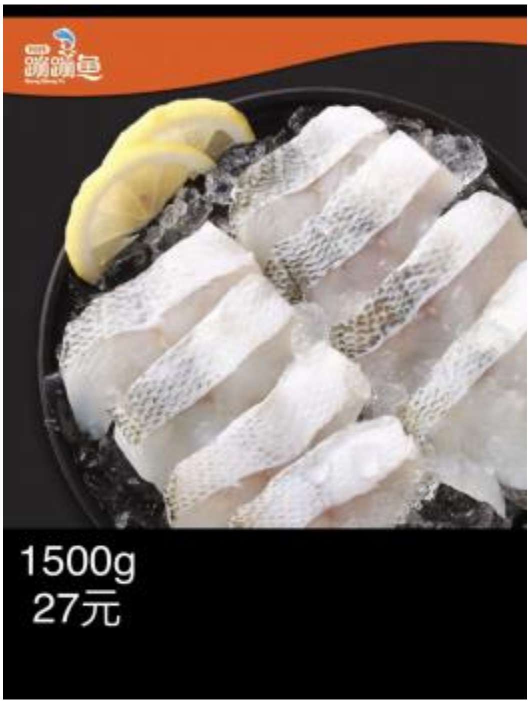	Name: sashimi<br>Calories：100kcal/100g<br>Protein：23.2g/100g<br>Fat：0.1g/100g

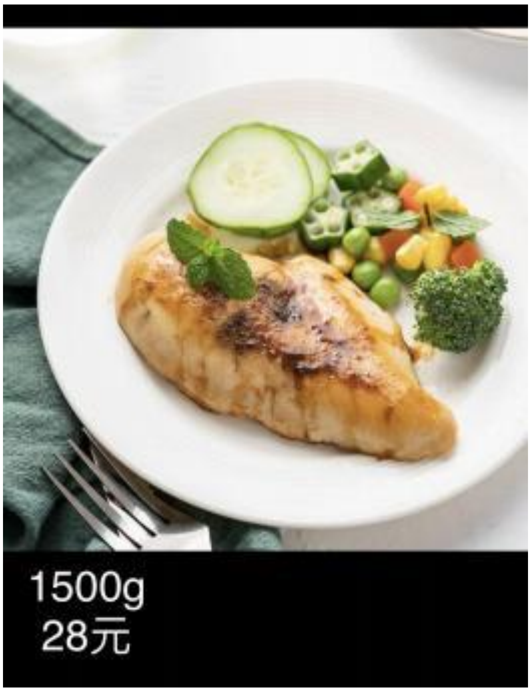	Name：fry chiken breast<br>Calories：110kcal/100g<br>Protein：27.6g/100g<br>Fat：1.6g/100g
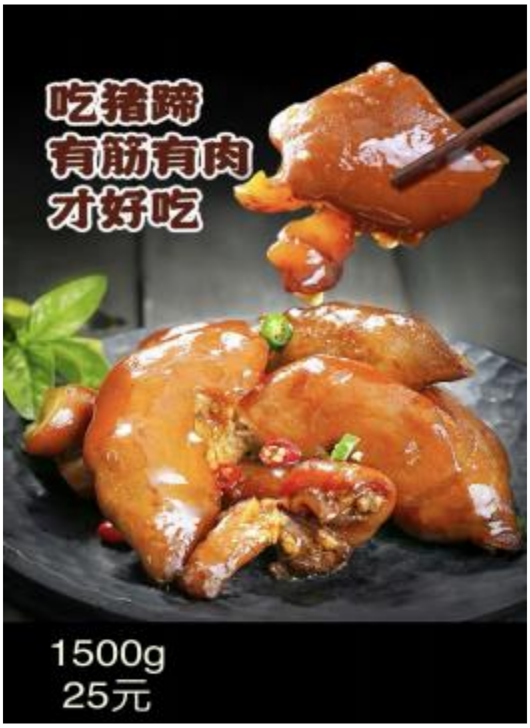	Name: simmered pig feet<br>Calories：237kcal/100g<br>Protein：28.4g/100g<br>Fat：15.4g/100g

Demographics

13. What’s your age?
14. What’s your gender?
15. What is your highest level of education so far?

o Bachelor
o Master
o PhD or above

16. What’s your monthly income (including contribution and scholarship) ?(RMB）
o Lower than 1000
o 1000 ~ 1500
o 1500 ~ 2000
o 2000 ~ 2500
o 2500 ~ 3000
o Above 3000

Thank you for participating in our survey. Your answer has been recorded.It is very much appreciated.

If you have any questions regarding the experiments or if you would like to receive the results of the survey you can contact us by j.li.32@student.rug.nl.

This is the end of the survey, thank you!
